# Enhanced Antibacterial Efficiency and Anti-Hygroscopicity of Gum Arabic–ε-Polylysine Electrostatic Complexes: Effects of Thermal Induction

**DOI:** 10.3390/polym15234517

**Published:** 2023-11-24

**Authors:** Ru-Yi Zhang, Peng-Fei Wang, Hua-Xiang Li, Yan-Jun Yang, Sheng-Qi Rao

**Affiliations:** 1 State Key Laboratory of Food Science and Technology, Jiangnan University, Wuxi 214122, China; 7230112055@stu.jiangnan.edu.cn (R.-Y.Z.); yangyj@jiangnan.edu.cn (Y.-J.Y.); 2 College of Food Science and Engineering, Yangzhou University, Yangzhou 225127, China; 18252789407@163.com (P.-F.W.); lihuaxiangyu@126.com (H.-X.L.)

**Keywords:** ε-polylysine, gum arabic, electrostatic complex, thermal induction, antibacterial activity, antihygroscopicity

## Abstract

The aim of this investigation was to scrutinize the effects of a thermal treatment on the electrostatic complex formed between gum arabic (GA) and ε-polylysine (ε-PL), with the goal of improving the antibacterial properties and reducing the hygroscopicity of ε-PL. The heated complex with a ratio of 1:4 exhibited an encapsulation efficiency of 93.3%. Additionally, it had an average particle size of 350.3 nm, a polydispersity index of 0.255, and a zeta potential of 18.9 mV. The formation of the electrostatic complex between GA and ε-PL was confirmed through multispectral analysis, which demonstrated the participation of hydrogen bonding and hydrophobic and electrostatic interactions, as well as the enhanced effect of heat treatment on these forces within the complex. The complex displayed a core-shell structure, with a regular distribution and a shape that was approximately spherical, as observed in the transmission electron microscopy images. Additionally, the heated GA–ε-PL electrostatic composite exhibited favorable antibacterial effects on Salmonella enterica and Listeria monocytogenes, with reduced minimum inhibitory concentrations (15.6 μg/mL and 62.5 μg/mL, respectively) and minimum bactericidal concentrations (31.3 μg/mL and 156.3 μg/mL, respectively) compared to free ε-PL or the unheated electrostatic composite. Moreover, the moisture absorption of ε-PL reduced from 92.6% to 15.0% in just 48 h after being incorporated with GA and subsequently subjected to heat. This research showed a way to improve the antibacterial efficiency and antihygroscopicity of ε-PL, reducing its application limitations as an antimicrobial substance to some extent.

## 1. Introduction

ε-polylysine (ε-PL) is a unique cationic polyamide derived from the extracellular material of filamentous bacteria or other eukaryotes. It consists of 25–30 l-lysine residues, and is connected by an amide bond between the α-carboxyl and ε-amino groups [1]. ε-PL has significant antibacterial ability, mainly due to its cationic property, as it has been reported that the mechanism of ε-PL’s antibacterial action involves breaking the cell membrane of bacteria through electrostatic adsorption with microorganisms that carry a negative charge [2]. It is also easily soluble in water, biodegradable, nontoxic, and resistant to thermal environments [3]. As a result, ε-PL exhibits excellent resistance to spoilage throughout various food processing and production stages, effectively restraining the growth of microorganisms and extending the shelf life of different food products. Nevertheless, the formation of insoluble precipitates can occur due to mutual attraction between the ε-PL cation and other anionic components present in the food system, leading to cloudiness in the solution system [4]. Moreover, its high hygroscopicity, tending to lead to aggregation, also makes it challenging to apply in practice. Therefore, one of the possible solutions to the problem is to manufacture polymer nano-delivery systems loaded with ε-PL to exert great antibacterial activity in the food system.

Polysaccharide, a natural polymer, is utilized in current research for encapsulation materials. Some examples of these polysaccharides include pectin [5], chitosan [6], and gum arabic [4]. Based on the positive charge characteristic of ε-PL, it dependably fabricates an electrostatic compound with an anionic polysaccharide via electrostatic interaction. Nevertheless, a complex based on electrostatic complexation tends to dissociate when external environmental conditions change [7]. Recent research has demonstrated that subjecting protein–polysaccharide electrostatic complexes to temperatures exceeding the protein’s denaturation point is vital for including intra/intermolecular conformational alterations, leading to improved particle stability and compactness of structure [8]. Thus, heating is widely used to strengthen hybrid nanostructures composed of polysaccharides and/or proteins. For instance, Dai et al. [9] reported a type of heated electrostatic complex consisting of a conjugate of whey protein isolate and dextran, along with chondroitin sulfate. This compound exhibited remarkable stability in various pH levels and salt ions found in environmental solutions. Gum arabic (GA) is a typical anionic hybrid polysaccharide rooted from the exudate of the branches or trunks of Acacia trees and a stable carrier to encapsulate and deliver bioactive substances via electrostatic complexation [10]. Additionally, it is important to mention that the containment of GA might have a role in safeguarding bioactive compounds against moisture interference. For example, there are reports indicating that the cranberry bush fruit powder, when encapsulated with gum arabic, exhibited reduced hygroscopicity compared to the nonencapsulated form. This characteristic enhances these substances’ stability throughout the storage period [11]. 

To our utmost knowledge, there exist no published studies elucidating the antibacterial attributes of the GA–ε-PL complex formed through ion crosslinking and heat simulation. Therefore, the present study aimed to (i) fabricate a GA–ε-PL electrostatic complex with heat treatment, (ii) investigate the physicochemical properties and structural characteristics of the heated GA–ε-PL electrostatic complex, and (iii) assess the antibacterial activity and moisture absorption capacity of the heated GA–ε-PL electrostatic complex as a natural environmentally friendly additive in food active packaging.

## 2. Materials and Methods

### 2.1. Materials

Two bacterial strains, *Salmonella enterica* CICC 21513 and *Listeria monocytogenes* ATCC 1911, were obtained from China Industrial Microorganism Species Conservation and Management Center. ε-PL was provided by Shanghai Macklin Biochemical Co., Ltd. (Macklin, Shanghai, China). The solid purity was greater than 95%, and the concentration of ε-PL in the aqueous solution was 10 mg/mL. GA was purchased from Sinopharm Chemical Reagent Co., Ltd. (Sinopharm, Shanghai, China). Methyl orange was obtained from Aladdin Biochemical Technology Co., Ltd. (Aladdin, Shanghai, China). All other chemicals utilized in this study were of analytical grade.

### 2.2. Preparation of the Heated GA–ε-PL Electrostatic Complex

**Scheme 1:**
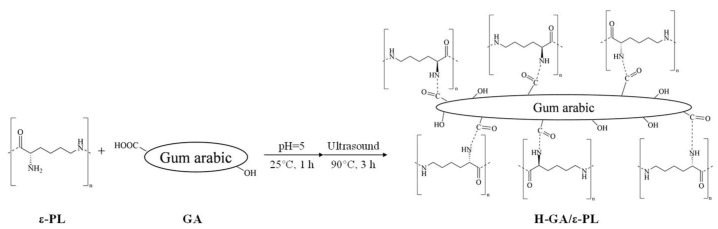
The chemical reaction of the heated GA–ε-PL electrostatic complex (H-GA/ε-PL).

The preparation of the heated GA–ε-PL electrostatic complex was based on the findings of Chang [4], with certain alterations. To obtain stock solutions of ε-PL and GA, 10 mg/mL of each compound was dispersed into deionized water. The pH was then adjusted to 5 using either HCl or NaOH solution. A 0.45 μm microporous membrane was used to remove insoluble material. To obtain a GA–ε-PL complex (GA/ε-PL), the solutions of ε-PL and GA were combined and agitated with a magnetic stirrer for 1 h at 25 °C to facilitate ion crosslinking. The examination of the electrostatic complex involved testing the effects of various preparation mass ratios of ε-PL and GA (ranging from 1:1 to 1:10) based on the current concentration of ε-PL at 0.5 mg/mL. Subsequently, a heated GA–ε-PL electrostatic complex (H-GA/ε-PL) was achieved by subjecting the aforementioned solution to a 90 °C water bath for 3 h and then dispersing it using ultrasound, as shown in Scheme 1.

### 2.3. Determination of Encapsulation Efficiency

The encapsulation efficiency of ε-PL was assessed using Liu’s method [12]. The sample solution underwent ultrafiltration by centrifugation at a speed of 10,000× *g* for a duration of 30 min. Next, 1 mL of the ultrafiltrate was combined with 4 mL of a solution containing methyl orange (0.5 mM). The mixture was then incubated at 30 °C for 30 min with vibration. Following this, the mixture solution underwent centrifugation at a force of 8000× *g* for 10 min, and the supernatant was diluted. The absorbance of the supernatant was determined at 465 nm using a UV-2550 UV–vis spectrophotometer (Shimadzu, Kyoto, Japan). The phosphate buffer functioned as a control with no effect. A 2 mg/mL ε-PL solution was prepared and subsequently diluted with deionized water to create a standard solution featuring mass concentrations of 0.05, 0.10, 0.15, 0.20, 0.30, 0.40, and 0.50 mg/mL, respectively. Then, 0.2 mL aliquots from the varied concentration standard solutions were utilized, supplementing each with 0.8 mL of a 0.5 mmol/L methyl orange solution. The next steps were as the same as mentioned above. A standard curve with concentration on the abscissa and OD_465_ on the ordinate was constructed. The standard curve for ε-PL was derived as y = 1.2588 − 1.84304x (R^2^ = 0.99066) (Figure S1). The calculation of the encapsulation efficiency of ε-PL was conducted using the following formula.

$$Encapsulation\;efficiency=\frac{C_{0}-C_{1}}{C_{0}}\times 100\%$$

*C*_0_ represents the initial concentration of ε-PL, while *C*_1_ indicates the ε-PL concentration of the measured supernatant.

### 2.4. Determination of Particle Dimensions and Zeta Potential

A laser particle sizer (DLS-5022F; Malvern, UK) was utilized to measure the size distribution and zeta potential through dynamic light scattering. Approximately 1 mL of sample (15%, *w*/*v*) was added to a measurement pool, called Marvin, with the temperature adjusted to 25 °C.

### 2.5. Intermolecular Force Analysis

The turbidity of the complex dispersoid was identified through the absorbance at 500 nm, conducted at 25 °C. The specimens were initially diluted at a 1:10 (*v*/*v*) ratio to maintain the linearity of the absorbance in the specified region. The measurement was conducted again to differentiate protein dispersoids containing sedimenting and nonsedimenting aggregates. The turbidity of samples, diluted in a solution containing 6 M urea, 0.5% sodium dodecyl sulfate (SDS), and 30 mM dithiothreitol (DTT), respectively, was assessed following a 10 min reaction period. This facilitated the evaluation of the intermolecular forces contributing to the establishment and sustenance of the complex structure.

### 2.6. Characterization of the GA–ε-PL Complex

#### 2.6.1. Fourier Transform Infrared (FTIR) Spectroscopy

FTIR spectroscopic analysis was carried out employing a Cary 610/670 FTIR spectrophotometric instrument (Varian, Palo Alto, CA, USA). After thorough drying, the samples were well mixed with KBr and then compressed into thin tablets. Afterwards, the dehydrated specimens and potassium bromide were finely ground and compacted into slender tablets. Furthermore, every spectrum underwent 64 scans at a resolution of 4 cm^−1^.

#### 2.6.2. X-ray Diffraction (XRD) Analysis

For the examination of crystal structures in the samples, a D8 Advance X-ray diffractometer (Bruker, Karlsruhe, Germany) was employed. A Cu target was utilized with an acceleration voltage of 40 kV and a current intensity of 200 mA. The counter utilized a sampling interval of 0.02°, while scanning at a speed of 0.15°/min within a 2θ range spanning from 2° to 60°.

#### 2.6.3. Differential Scanning Calorimeter (DSC) Analysis

The determination of sample formation was executed utilizing a DSC 8500 differential scanning calorimeter (Perkin Elmer, Shelton, CT, USA). Before measurements were taken, zinc and indium were used to calibrate the instrument. The analysis was conducted at a scanning rate of 10 °C/min, commencing at 25 °C and concluding at 120 °C. All DSC measurements were conducted in a nitrogen atmosphere at a flow rate of 20 mL/min.

#### 2.6.4. Microstructure Analysis

Transmission electron microscopy (TEM) analysis was conducted using a Tecnai 12 (Philips, Eindhoven, The Netherlands) at an acceleration voltage of 100 kV. The samples were placed onto a copper grid and stained using an aqueous stain containing 1.5% phosphotungstic acid for 1 min, followed with the absorption of the excess staining solution.

### 2.7. Antibacterial Capability Evaluation

#### 2.7.1. Minimum Inhibitory Concentration (MIC) and Minimum Bactericidal Concentration (MBC)

The MICs of *S. enterica* and *L. monocytogenes* were measured using Zhao’s broth dilution method, with certain adjustments [13]. Two bacteria strains were cultivated in LB broth at 37 °C for a duration of 24 h. Following this, the bacterial suspensions were appropriately diluted to a concentration of 1 × 10^5^ CFU/mL during the exponential growth phase. The gradient samples were meticulously prepared employing the double dilution method with LB medium. The bacterial suspensions were added to a variety of samples and incubated at 37 °C for a duration of 24 h. MIC stands for the minimal concentration of a clarified sample contained in the test tubes. A negative control was established by setting up media without adding antibacterial samples, with an equal volume of sterilized PBS. To determine the MBCs, the suspensions extracted from the clarified sample tubes, which were employed in the MIC test, were inoculated onto LB agar plates and subsequently incubated at 37 °C for a duration of 24 h. The MBC is defined as the lowest concentration of the sample within the test tube plated on the agar, where bacterial growth is conspicuously absent. 

#### 2.7.2. Time-Dependent Inhibition Curves

The antibacterial efficiency of the samples against *S. enterica* and *L. monocytogenes* was assessed by measuring the time-dependent inhibition curves, following the method of Rao [14], with minor adjustments. The concentration of the bacterial suspensions was modified to 5 × 10^5^ CFU/mL using LB medium. Afterwards, the samples were included with an equivalent concentration to 1 MIC heated GA–ε-PL electrostatic complex and then incubated at 37 °C. The bacterial counts were measured every 2 h within 24 h. A testing group with equal bacterial dilution was set up as a negative control. 

### 2.8. Hygroscopicity Assay

The assessment of moisture absorption was conducted using Hazaveh’s static gravimetric method [15], with certain alterations. The environmental temperature and the relative humidity of the glass desiccator were respectively maintained at around 25 °C and 60%. Afterwards, the pre-weighed samples were placed into the desiccator and weighed after different storage times (12, 24, 36, and 48 h). The moisture-absorption rate was calculated as follows:

(1) $$Moisture-absorptionrate=\frac{M_{n}-M_{0}}{M_{n}}\times 100\%$$

*M*_0_ represented the weight of initial samples, while *M_n_* denoted the weight of samples following various durations of storage.

### 2.9. Statistical Analysis

All samples were collected in triplicate, and the experimental data are expressed as the mean and standard deviation of the three measurements. SPSS software 22 was utilized to conduct statistical analyses employing analysis of variance.

## 3. Results and Discussion

### 3.1. Complex Formation

**Figure 1:**
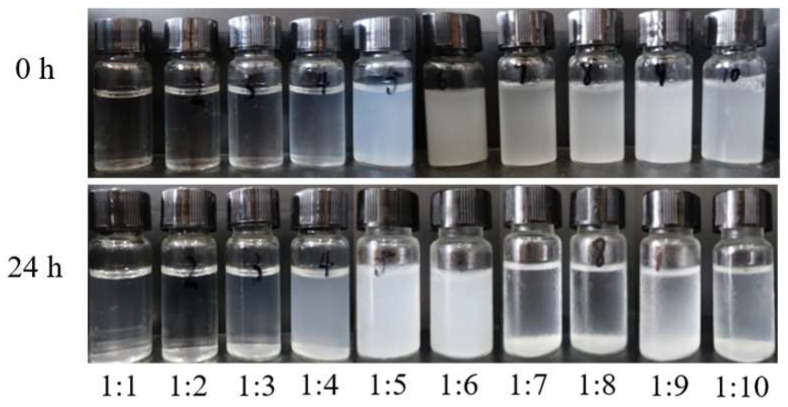
The visual appearance of gum arabic–ε-polylysine (GA–ε-PL) electrostatic complexes with different ratios of ε-PL:GA (1:1–1:10) before/after standing for 24 h.

The preliminary assessment of the stability of the water solution composites of ε-PL and GA was conducted by visually observing turbidity. The turbidity and appearance of the solution at pH 5.5 were used to observe a range of mixed solutions containing ε-PL (0.5 mg/mL) and varying concentrations of GA (0 mg/mL–5 mg/mL). To ensure a uniform distribution, it was necessary to thoroughly mix the samples before measuring turbidity. Before visual observation, the samples were also allowed to stand at 25 °C for 24 h to evaluate the stability of the sample solutions under gravity delamination. As depicted in Figure 1, the stability of GA and ε-PL significantly differed when the mass ratio varied. Initially, following the increase in the addition ratio of GA:ε-PL, the turbidity observed visually raised considerably. Notably, when the mass ratio of GA:ε-PL exceeded 6:1, the complex precipitated after 24 h. As we know, tiny soluble substances cannot scatter light intensely, resulting in the constitution of clarified solutions with low turbidity. On the contrary, the light is powerfully scattered by big, insoluble substances, and, thus, highly turbid colloidal suspensions with a certain amount of sediment could be formed [16]. Thus, this phenomenon could be ascribed to the electrostatic complexation of cationic ε-PL and anionic GA, and the same case was also observed in Chang’s reports [4]. However, when the GA concentration was higher, the formation of complex particles was larger, resulting in aggregation and precipitation. Therefore, in subsequent experiments, material ratios of ε-PL:GA in the range from 1:1 to 1:5 were selected to avoid precipitation.

The aforementioned findings indicate that an ionic crosslinking reaction occurred between ε-PL and GA. To ascertain the optimal preparation parameters, it is imperative to investigate the influence of different addition ratios of ε-PL and GA, ranging from 1:1 to 1:5, on the characteristics of unheated and heated electrostatic complexes. This evaluation involved contradistinguishing the average particle size, PDI, zeta potential, and encapsulation efficiency. As exhibited in Table 1, the electrostatic composite at mass ratios of 1:1 and 1:2 cannot have its average diameter and PDI measured. This could be due to the absence of enough GA, which could form particle states capable of being measured. Subsequently, there was a notable rise in the average particle size of the unheated GA–ε-PL electrostatic complex as the mass ratio decreased from 1:3 to 1:5, while the heated complex exhibited a similar trend within the mass ratio range of 1:1 to 1:5. The findings can be attributed primarily to the presence of ample GA encapsulation on the ε-PL surface, and a similar occurrence was noted in nanoparticles loaded with rosemary extract [17]. Most notably, the heated electrostatic complex’s mean particle size was larger overall than that of the unheated one at the same mass ratio. This could be explained by the quantity of small molecules that existed in the unheated complex and then aggregated after heat treatment, resulting in a significant augmentation in particle size, similar to the result of Loveday [18]. It is common knowledge that PDI serves as a crucial measure of particle dispersibility, and a PDI value lower than 0.3 indicates excellent polydispersion [19]. It was observed that the PDIs of heated GA–ε-PL electrostatic complexes at mass ratios of 1:4 and 1:5 were below 0.3, suggesting a narrow size distribution and great dispersibility and uniformity.

Zeta potential (ZP) is a significant parameter that could examine the electrodynamics performance of charged particles [12]. According to the information presented in Table 1, ZP analyses were conducted to examine the charge of the complex and the electrostatic interactions between the positively charged ε-PL and negatively charged GA. With the increasing addition of GA, the ZPs of the unheated and heated electrostatic complexes decreased gradually and remained in the positive range from 10 to 30 mV. This should be attributed to the negative charge and charge shielding against cationic ε-PL provided by anionic GA on the surface of the complex, similar to the result of Liu [19]. In addition, it was observed that compared to the ZPs of unheated complexes, those of the heated complexes are generally higher at different mass ratios of ε-PL and GA. This is favorable evidence of the enhancement of the adsorption of more GA and the electrostatic binding after heat treatment. This tendency was consistent with the result reported by other authors for potato protein–GA electrostatic complexes after heat induction [20].

Certainly, by reducing the mass ratio from 1:1 to 1:5, the encapsulation efficiency (EE) of both unheated and heated electrostatic complexes increased gradually. Significantly, when the proportion of ε-PL to GA exceeded 1:3, the efficiency of encapsulating ε-PL molecules in the unheated electrostatic complex dropped below 90.47%, indicating that the GA shell was insufficient to fully capture all ε-PL molecules. On the other hand, when the mass ratio was below 1:3, the EE was increased from 90.47% (1:3) to 94.92% (1:5). This result suggested that most of the ε-PL molecules were successfully encapsulated in the GA shell. The same phenomenon was also observed in curcumin-loaded rhamnolipid nanoparticles [21]. Additionally, the EEs of the heated electrostatic complexes were enhanced to a high level in the range of 88.54–97.74% compared with those of the unheated complexes. This result should be attributed to the strengthening of intermolecular forces after heat treatment, which could make ε-PL difficult to dissociate from the GA shell, resulting in enhanced system stability. Comparatively, Pieczykolan [22] reported that the EE of GA microcapsules loaded with anthocyanins was only 78.61%, far below that of our reports. Additionally, the EE of the heated electrostatic complex was also higher than previous reports of bioactive-substance-loaded GA-based encapsulation systems [23,24,25].

The above findings suggest that ε-PL and GA formed a stronger bond as a result of the robust intermolecular interaction following the application of heat. Considering the thorough assessment of the particle size, PDI, zeta potential, and encapsulation efficacy of the heated electrostatic complexes, it is plausible to contemplate the mass ratio of ε-PL to GA as 1:4 or 1:5. In addition, taking into account that a smaller size of particles and a higher zeta potential are favorable for the manifestation of subsequent antibacterial effects, the best ratio of ε-PL to GA mass for the production of a heated electrostatic complex is 1:4.

### 3.2. Intermolecular Force Analysis

**Figure 2:**
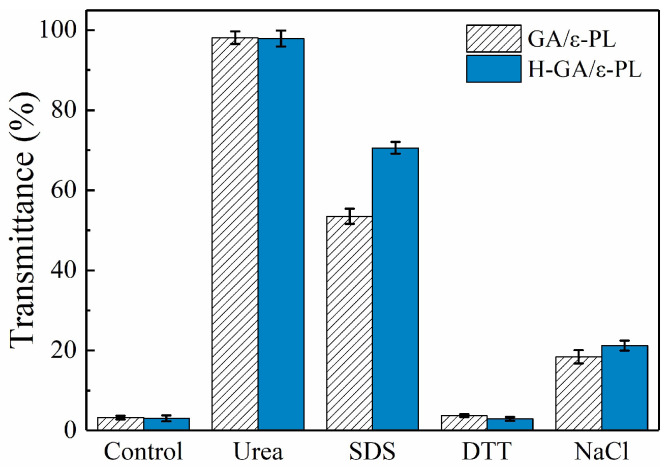
Intermolecular forces analysis of the unheated GA–ε-PL electrostatic complex (GA/ε-PL) and heated GA–ε-PL electrostatic complex (H-GA/ε-PL).

The colloidal stability of the heated electrostatic complex was examined by turbidity value (Figure 2). By dissolving or diluting the sample with a high-concentration NaCl solution, the electrostatic interaction in the solution can be reduced, and the charge effect can be shielded or reduced [26]. The structure of water molecules can be affected by urea, leading to interference with the formation of hydrogen bonds [27]. SDS can also affect the structure of water molecules, but SDS prevents hydrophobic interactions [28]. DTT can reduce and influence the formation of intermolecular disulfide bonds [27]. In the absence of a denaturant, the turbidity of the unheated and heated electrostatic complexes was relatively high, and both light transmittances reached only about 3%. After the addition of DTT, both unheated and heated electrostatic complexes hardly changed, indicating that the formation of the microgranular texture was unrelated to intermolecular disulfide bonds. However, for the unheated complex, the transmittance of the NaCl, SDS, and urea treatment groups significantly raised to 58.4%, 18.5%, and 98.1%, respectively. These results indicated that the main intermolecular forces of the unheated complex were electrostatic interaction, hydrophobic interaction, and hydrogen bonds, which played a dominant role; the trend of the heated electrostatic complex was the same. On this basis, the transmittance of the heated electrostatic complex in the SDS and NaCl treatment groups increased to 70.6% and 21.2%, respectively. It could be speculated that heating could, to some extent, promote the part formation of intermolecular interaction, which is supported by Jones and McClements [8]. It has been proposed that heating promotes the hydrophobic interaction, leading to the decrease in solubility and the decomposition of aggregation [9].

### 3.3. Structure Analysis of the GA–ε-PL Complex

#### 3.3.1. FTIR Analysis

**Figure 3:**
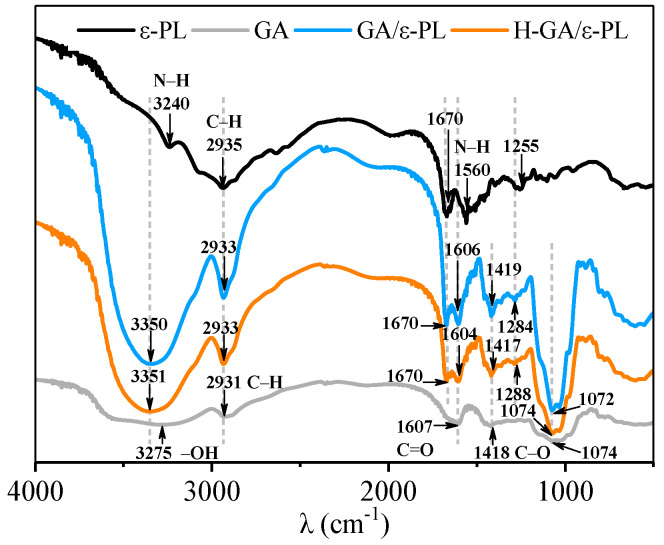
Fourier transform infrared (FTIR) spectra of ε-PL, GA, and unheated and heated GA–ε-PL electrostatic complexes.

The complex’s intermolecular interaction and structure were determined using FTIR analysis. Figure 3 displays the FTIR spectra of the electrostatic complexes formed by ε-PL and GA, both unheated and heated. In the ɛ-PL spectrum, the N–H symmetrical stretching vibration was observed at 3240 cm^−1^, while the C–H stretching vibration was observed at 2935 cm^−1^, representing the usual peaks [6]. GA’s spectrum exhibited distinct peaks at 3275 cm^−1^, indicating –OH stretching, 2931 cm^−1^ for symmetric and asymmetric C–H vibration, 1601 cm^−1^ for C=O stretching, and 1418 cm^−1^ and 1074 cm^−1^ for C–O stretching. These observations align closely with those documented in the existing literature [29,30]. The FTIR spectra of the unheated and heated electrostatic complexes exhibited a displacement in the N–H stretching peak of ε-PL (3240 cm^−1^) and O–H stretching of GA (3275 cm^−1^) towards a higher wavenumber of 3350 cm^−1^ and 3351 cm^−1^, respectively. The occurrence of this phenomenon can be credited to the creation of fresh robust hydrogen bonds between the O–H stretch of GA and the N–H groups of ε-PL. The peak of C–H stretching vibration in ε-PL spectra (2935 cm^−1^) and GA spectra (2931 cm^−1^) changed to 2933 cm^−1^ for both the unheated and heated electrostatic complexes. The intensity of the peak in the GA–ε-PL complex at 1670 cm^−1^ also diminished. Moreover, in the spectra of the GA–ε-PL complex, there was a slight shift observed at 1607 cm^−1^, 1418 cm^−1^, and 1074 cm^−1^ in the stretching vibration of the carboxyl group associated with GA. The presence of electrostatic interactions between charged amino groups in ε-PL and electronegative COO− groups of GA is likely responsible for these observed modifications. Furthermore, the N–H bending peak in the ε-PL spectra showed a shift from 1560 cm^−1^ to 1606 cm^−1^ and 1604 cm^−1^ in the GA–ε-PL electrostatic complex, both before and after heating. The alterations in the peaks of the secondary N–H bending validated the spatial modifications of the ε-PL configuration, leading to the revelation of additional hydrophobic clusters and enhancement of hydrophobic connections between ε-PL and GA [31]. The spectra of the unheated and heated electrostatic complexes showed that the amide I and amide II peaks were higher in the unheated complex compared to the heated complex. The formation of additional hydrophobic and electrostatic interactions between ε-PL and GA after heating in the GA–ε-PL complex is responsible for this outcome, as confirmed by the findings of intermolecular force analysis (Figure 2). Many other studies also reported the enhancement of intermolecular interactions after heat treatment between biopolymers [7,31]. In total, the formation of unheated and heated GA–ε-PL complexes was driven by hydrogen bonding and hydrophobic and electrostatic interactions, and heating strengthened these forces within the complexes.

#### 3.3.2. XRD Determination

**Figure 4:**
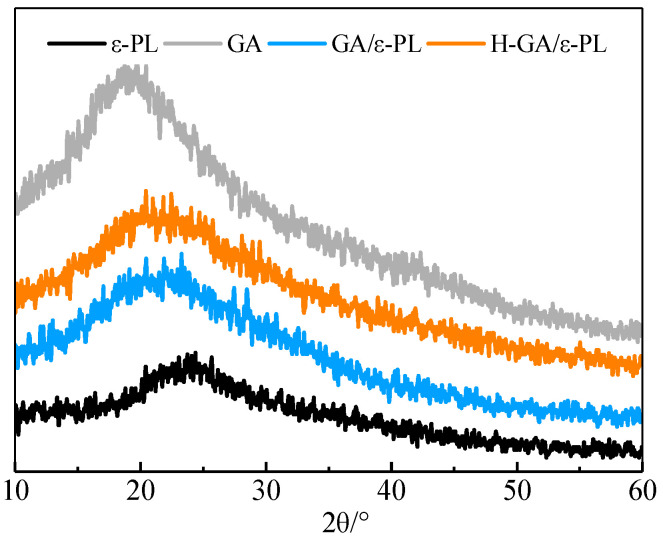
X-ray powder diffraction (XRD) spectra of ε-PL, GA, and unheated and heated GA–ε-PL electrostatic complexes.

As exhibited in Figure 4, the crystalline structures of the samples were evaluated. The pattern of ε-PL showed a wide peak between 20° and 27°, indicating its amorphous structure, consistent with the findings of Jia et al. [32]. For GA powder, the broad peak at 2θ = 18.79° revealed the existence of a semi-crystalline phase, similar to previous results [33]. In addition, the GA–ε-PL complex showed distinct peaks at 2θ = 21.82° and 20.44° for the unheated and heated samples, respectively. These characteristic peaks indicated that ε-PL and GA were not simply mixed. The addition of ɛ-PL to GA was found to decrease crystallinity, as evidenced by the reduction in peak intensity and the emergence of wider peaks. The reason behind this occurrence could be explained by the rearrangement of organized formations of the GA–ε-PL complex caused by the inclusion of ɛ-PL [34]. Moreover, no new characteristic peaks were formed in the XRD spectra, proving that no new crystal structure was formed. To summarize, there was evidence of intermolecular interaction between ε-PL and GA, resulting in the successful formation of the GA–ε-PL complex. 

#### 3.3.3. DSC Assay

**Figure 5:**
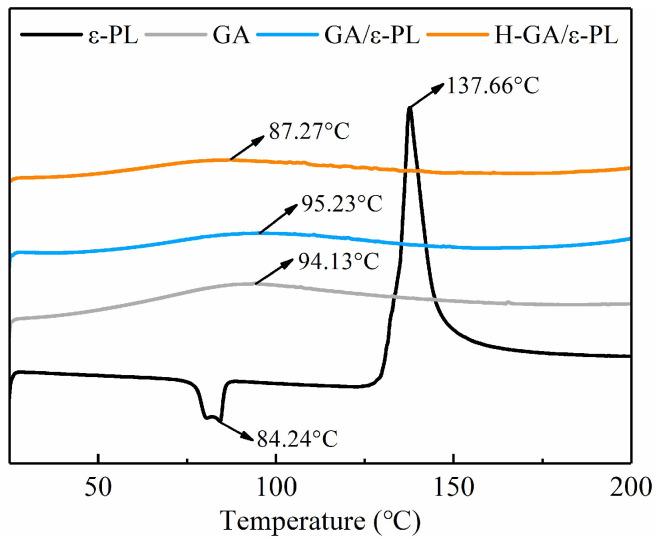
Differential scanning calorimetry (DSC) spectra of ε-PL, GA, and unheated and heated GA–ε-PL electrostatic complexes.

As depicted in Figure 5, DSC was used to analyze the thermal characteristics of the complex. The initial endothermal peak of ε-PL at 84.24 °C was ascribed to the vaporization of the attached moisture, resembling the finding of Zhang [35] who documented the endothermal peak at 89.7 °C. Furthermore, ε-PL exhibited a sharp exothermic peak at 137.66 °C, indicating its melting point. The GA’s DSC thermogram showed a broad characteristic endothermic peak at 94.13 °C, which was attributed to the evaporation of water. This result was consistent with the research of Sabet, who reported that the endothermic peak was close to 100 °C for GA [36]. Significantly, the DSC thermographs of the unheated and heated GA–ε-PL complexes exhibited a greater resemblance to GA, since the ε-PL hot-melt peak vanished. The reason behind this occurrence can be ascribed to the encapsulation of ε-PL within GA, which acted as a core and facilitated the creation of amorphous complexes. The unheated complex exhibited a slightly higher peak temperature (95.23 °C) compared to the heated complex (87.27 °C), as observed. The increase in the hot-melt peak temperature may be attributed to the incomplete integration of a portion of ε-PL.

#### 3.3.4. TEM Observation

**Figure 6:**
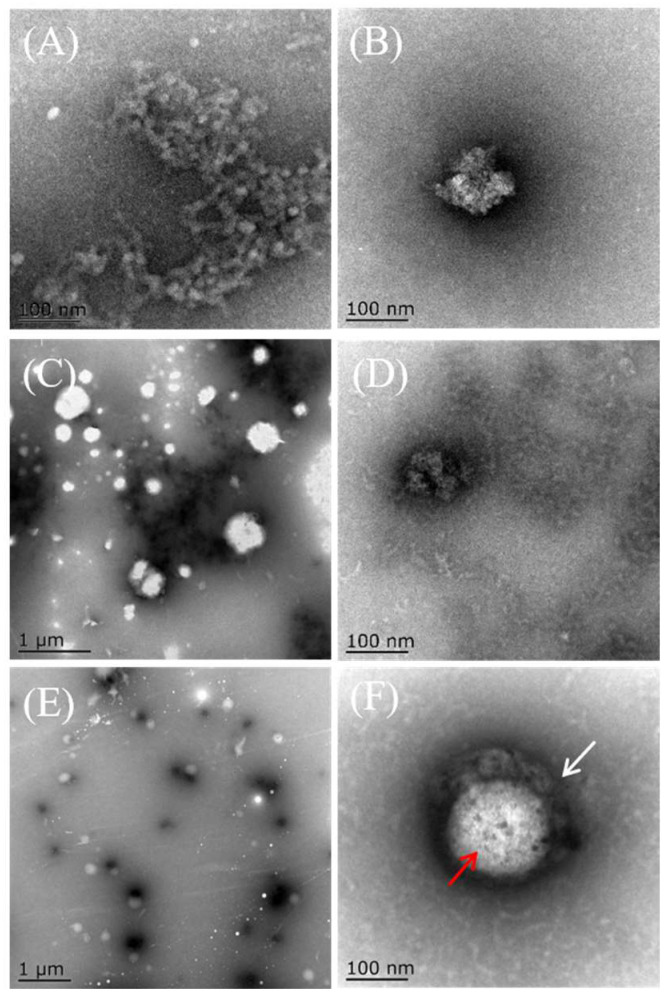
Transmission electron microscopy (TEM) images of ε-PL (**A**), GA (**B**), the unheated GA–ε-PL electrostatic complex (**C**,**D**), and the heated GA–ε-PL electrostatic complex (**E**,**F**). The white arrow indicates the shell of particle, and the red arrow indicates the core of particle.

TEM was used to analyze the microstructure of the samples. As depicted in Figure 6, ε-PL (Figure 6A) and GA (Figure 6B) were able to be dissolved in water and had irregular shapes, such as flocculate and block. The untreated GA–ε-PL complex (Figure 6C,D) had irregular morphology and rough surface, and part of the ε-PL was only attached to the surface of the GA via electrostatic interaction, showing a relatively lower polymerization degree. After heat treatment (Figure 6E,F), the size of the complex increased from about 100 nm to 200 nm, and the binding state became more stable. The reason for this can be credited to the fact that the electrostatic interaction became strong following the thermal treatment, causing ε-PL to separate and be released from GA, ultimately forming irreversible aggregates in the surrounding solution. Subsequently, after the temperature dropped, the ε-PL molecules, which had positive charges on their surfaces, were still capable of binding with negatively charged GA. This led to the creation of a complex with a core-shell structure, similar to the phenomenon observed in heat-treated electrostatic β-lactoglobulin–pectin complexes mentioned in previous studies [37]. As depicted in Figure 6F, the core-shell arrangement was easily identifiable where the shell (indicated by white arrows) exhibited a higher electron density in comparison to the core (indicated by red arrows). Therefore, the above results indicated that the heated complex could well encapsulate ε-PL through thermal induction. 

### 3.4. Antibacterial Properties

**Figure 7:**
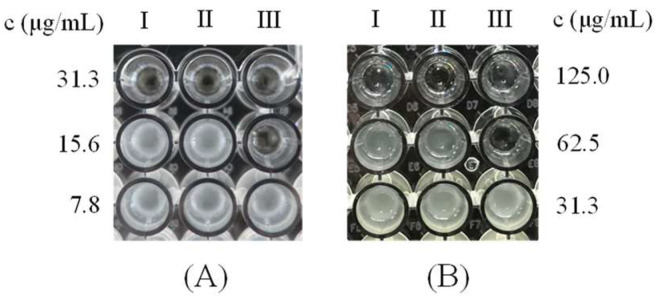
Images of ε-PL (I), the unheated GA–ε-PL electrostatic complex (II) and the heated GA–ε-PL electrostatic complex (III) on the inhibition of bacteria ((**A**) *Salmonella enterica*; (**B**) *Listeria monocytogenes*) in incubating wells.

Figure 7 shows images of samples on the inhibition of bacteria in incubating wells. Apparently, the bacteria incubating wells treated with ε-PL and the unheated GA–ε-PL electrostatic complex became turbid at the concentrations of 15.6 μg/mL (for *S. enterica*) and 62.5 μg/mL (for *L. monocytogenes*). Surprisingly, the heated GA–ε-PL electrostatic complex can lower their turbidity threshold by half. Table 2 displays the bacteriostatic characteristics of the GA–ε-PL complexes, whether heated or unheated, against *S. enterica* and *L. monocytogenes*. GA lacks antimicrobial properties, while ε-PL demonstrates noteworthy antimicrobial efficacy against a wide array of both gram-negative and gram bacteria owing to its positively charged amino acids [35]. The MICs and MBCs of the unheated complex against both bacteria were identical to those of ε-PL, suggesting that the sample obtained solely through ion crosslinking did not enhance the antibacterial efficacy of ε-PL. Nevertheless, the heated complex demonstrated greater bacteriostatic effects as evidenced by a twofold decrease in both the MIC and MBC compared to ε-PL and the untreated complex. This result should be ascribed to the more abundant ε-PL encapsulated in GA after thermal treatment. Furthermore, encapsulation of chitosan-sodium alginate nanoparticles provided ε-PL with long-lasting antibacterial effects [12].

In addition, the MICs and MBCs of the complex varied for the two strains due to their distinct cellular compositions. It is well-known that ε-PL has the ability to adhere to the surface of cells by electrostatic absorption, resulting in the disruption of the cell wall structure and the physical breakdown of the cell membrane. This can lead to the release of internal cytoplasm and, ultimately, result in cell death [3]. In this case, the complex presented more effective antibacterial activities on *S. enterica* (gram-negative) compared to *L. monocytogenes* (gram-positive) due to the thinner peptidoglycan layer, which was easy to destroy. The same phenomenon was also observed in the ε-PL-loaded chitosan–sodium alginate complex [12] and ε-PL modified with different reducing sugars [1].

**Figure 8:**
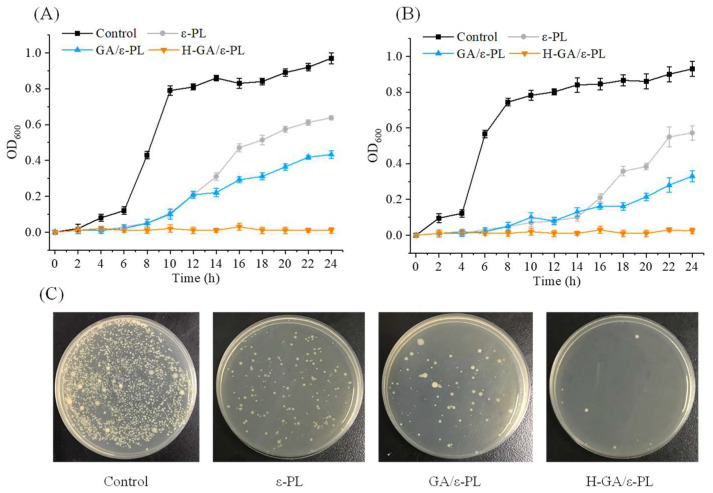
Effects of ε-PL and unheated and heated GA–ε-PL electrostatic complexes on strain growth: (**A**) *Salmonella enterica* and (**B**) *Listeria monocytogenes*; (**C**) Effects of different samples on bacterial growth on plate medium.

Figure 8 demonstrates the impact of the GA–ε-PL complex, whether heated or unheated, on the growth of bacterial strains, which were used to reflect the antibacterial activity of the complex. Free ε-PL and the complex exhibited significant antimicrobial activity against both strains in comparison with the control groups, with ε-PL playing a prominent role at the same concentration. Moreover, it was noted that the bacteria exposed to unbound ε-PL and the non-heated GA–ε-PL complex initiated their growth approximately 12 h later. Conversely, the bacteria treated with the heated complex remained in a non-proliferative state, suggesting that the long-lasting antibacterial properties of the heated GA–ε-PL complex surpassed those of the unbound ε-PL and non-heated complex. The reason for this outcome can be attributed to the close relationship between the inhibitory activity of ε-PL on microorganisms and its polymerization degree [38]. As time progresses, ε-PL may undergo dissociation in solution, resulting in a decrease in polymerization degree, which in turn leads to a decline in antibacterial effectiveness and an increase in bacterial growth. Most notably, GA encapsulation could maintain the high degree of aggregation of ε-PL, resulting in the extension of its inhibitory effect on bacteria. Furthermore, heat treatment may strengthen intermolecular interaction to stabilize the protein–polysaccharide structure and promote the complete encapsulation of plentiful ε-PL, contributing to the subsequent long-acting release [8]. In addition, *S. enterica* treated with different bacteriostatic agents was taken out and plated after incubation for 12 h. As shown in Figure 8C, the reduced colony densities and colony numbers can also prove the enhanced bacteriostatic effect of H-GA/ε-PL.

### 3.5. Moisture-Absorption Characteristics

**Figure 9:**
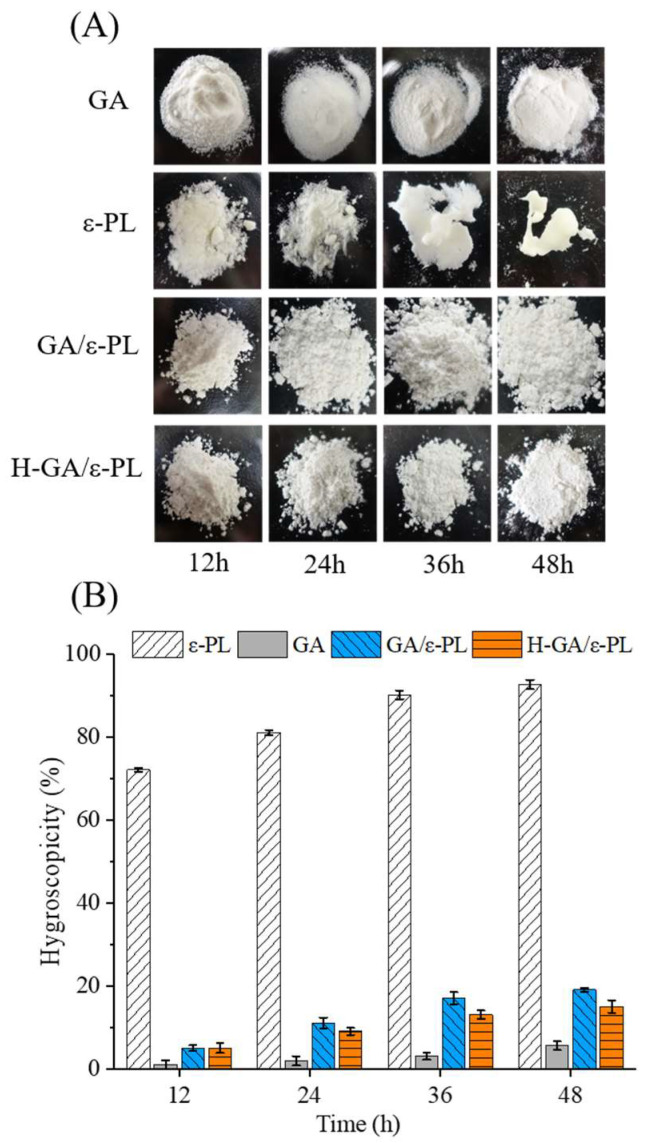
The powder appearance images (**A**) and hygroscopic changes (**B**) of ε-PL, GA, and unheated and heated GA–ε-PL electrostatic complexes.

Figure 9 shows the hygroscopicity changes of ε-PL, GA, and the unheated and heated GA–ε-PL complexes. It is reported that it is advisable to reduce hygroscopicity to preventing agglomeration because the caking may lead to the degradation of bioactive substances and the obstruction of dispersity, which could subsequently hinder further application [39]. Indeed, every sample showed an increasing trend of hygroscopicity within 48 h. Most notably, the ε-PL powder had the highest hygroscopicity from 72.0% to 92.6% (Figure 9B). From Figure 9A, it is also easily observed that following the increase in moisture absorbed, the ε-PL powder tended to agglomerate, leading to the formation of a blocky structure with a smooth surface. Thus, the strong hygroscopicity of ε-PL prevents it from being used alone; therefore, it is necessarily mixed with other substances to ensure stability in its application, such as pectin [40], chitosan [41], and starch [35]. On the contrary, the GA powder had a strong resistance to hygroscopicity. The variation in hygroscopic values arises from the properties of powders and their ability to absorb moisture from the surrounding atmosphere. This absorption rate is influenced by the presence of hydrophilic groups in each type of carrier [42]. Additionally, it was noted that the moisture-absorbing property of the GA–ε-PL complexes, both unheated and heated, was observed (Figure 5B) to be no more than 20%. The rationale for this phenomenon can be ascribed to the fact that GA effectively enveloped ε-PL, significantly reducing its exposure to air moisture and consequently leading to a substantial decline in ε-PL’s hygroscopic nature. Furthermore, the hygroscopicity of the unheated complex was slightly higher than that of the heated one. The decrease in polar interactions with water can be attributed to the disruption of hydrogen bonds and the decrease in hydrophilic groups caused by the heating process [39,42].

## 4. Conclusions

The core-shell structural electrostatic complexes of GA and ε-PL were successfully obtained by ionic crosslink and heat induction. The complex obtained under the ideal ratio condition of ε-PL:GA (1:4) exhibited a significant EE of 93.3%, with an average particle size measuring 350.3 nm. Additionally, it displayed a ZP of 18.9 mV and a PDI of 0.255. According to the FTIR, XRD, DSC, and TEM analyses, the encapsulation of GA resulted in a remarkable fusion of ε-PL with the particle wall. Compared to the unheated complex and free ε-PL, the heated GA–ε-PL electrostatic complex exhibited improved antibacterial efficacy and reduced hygroscopicity. The above findings provide evidence that the combination of ε-PL and GA holds promise for utilization in the food industry as a viable substitute for conventional chemical preservatives.

## Figures and Tables

**Table 1:** Effects of mass ratio on particle size, PDI (polydispersity), zeta potential, and encapsulation efficiency of unheated GA–ε-PL electrostatic complexes (GA/ε-PL) and heated GA–ε-PL electrostatic complexes (H-GA/ε-PL).

Sample	Mass Ratio (ε-PL:GA)	Particle Size (nm)	PDI	Zeta Potential (mV)	Encapsulation Efficiency (%)
GA/ε-PL	1:1	n.d.	n.d.	25.10 ± 1.01 ^b^	83.74 ± 1.66 ^f^
	1:2	n.d.	n.d.	20.60 ± 0.79 ^cd^	82.88 ± 1.40 ^f^
	1:3	170.80 ± 13.79 ^e^	0.58 ± 0.10 ^b^	19.30 ± 0.37 ^d^	90.47 ± 1.20 ^d^
	1:4	194.90 ± 12.35 ^d^	0.35 ± 0.07 ^c^	15.00 ± 0.47 ^e^	91.88 ± 0.72 ^cd^
	1:5	255.00 ± 14.55 ^c^	0.47 ± 0.06 ^bc^	12.80 ± 0.53 ^f^	94.92 ± 0.78 ^b^
H-GA/ε-PL	1:1	126.00 ± 14.77 ^f^	0.86 ± 0.19 ^a^	27.00 ± 0.66 ^a^	88.84 ± 0.66 ^e^
	1:2	199.00 ± 63.74 ^d^	0.80 ± 0.21 ^a^	22.00 ± 0.59 ^c^	90.27 ± 0.99 ^d^
	1:3	277.30 ± 22.59 ^c^	0.53 ± 0.22 ^b^	23.60 ± 0.60 ^bc^	95.64 ± 1.10 ^b^
	1:4	350.30 ± 5.95 ^b^	0.26 ± 0.04 ^c^	18.90 ± 1.03 ^d^	93.29 ± 0.83 ^c^
	1:5	465.60 ± 9.74 ^a^	0.18 ± 0.01 ^d^	16.40 ± 0.61 ^e^	97.74 ± 1.10 ^a^

Data are mean values ± standard deviation. Values with different letters in each column indicate significances different at *p* < 0.05. n.d. means not detected.

**Table 2:** Minimum inhibitory concentration (MIC) and minimum bactericidal concentration (MBC) of ε-PL and unheated and heated GA–ε-PL electrostatic complexes.

Strains	MIC (μg/mL)		MBC (μg/mL)	
	*S. enterica*	*L. monocytogenes*	*S. enterica*	*L. monocytogenes*
ε-PL	31.3 ± 2.33 ^a^	125.0 ± 8.41 ^a^	62.5 ± 6.51 ^a^	312.5 ± 12.17 ^a^
GA/ε-PL	31.3 ± 4.82 ^a^	125.0 ± 6.98 ^a^	62.5 ± 7.19 ^a^	312.5 ± 9.84 ^a^
H-GA/ε-PL	15.6 ± 1.51 ^b^	62.5 ± 4.12 ^b^	31.3 ± 3.75 ^b^	156.3 ± 8.73 ^b^

Data are mean values ± standard deviation. Values with different letters in each column indicate significances different at *p* < 0.05.

## Data Availability

Data are contained within the article.

## Supplementary Materials

The following supporting information can be downloaded at: https://www.mdpi.com/article/10.3390/polym15234517/s1, Figure S1: The standard curve for ε-PL.
