# Supplementary figures and images for: Enhanced Antibacterial Efficiency and Anti-Hygroscopicity of Gum Arabic–ε-Polylysine Electrostatic Complexes: Effects of Thermal Induction
Source: Polymers (Basel). 2023 Nov 24;15(23):4517. doi: 10.3390/polym15234517 (PMC10708452; doi:10.3390/polym15234517)

### Supplementary Materials

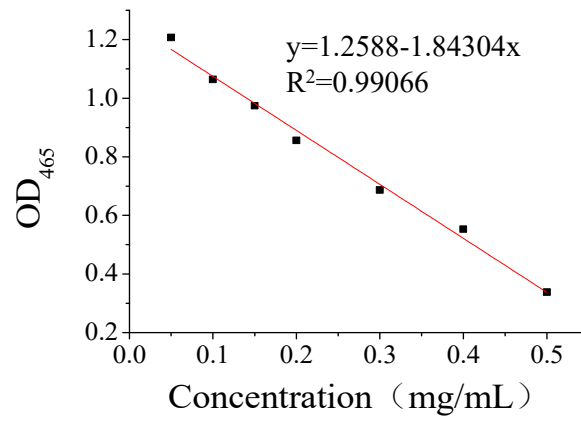

**Figure S1.** The standard curve for ε-PL.

Supplement: Supplementary file 1 [file polymers-15-04517-s001.zip › polymers-2690745-supplementary.pdf]
